# Progressive dyspnea in patient with large mediastinal mass

**DOI:** 10.1186/1749-8090-9-6

**Published:** 2014-01-06

**Authors:** Shinichi Fukuhara, Kamellia R Dimitrova, Charles M Geller, Darryl M Hoffman, Wilson Ko, Robert F Tranbaugh

**Affiliations:** 1 Division of Cardiac Surgery, Beth Israel Medical Center, 317 East 17th Street, 11th Floor, New York, NY 10003, USA

**Keywords:** Primary mediastinal neoplasm, Liposarcoma, Surgical resection

## Abstract

Liposarcoma occurs very rarely in the mediastinum. Patients often remain asymptomatic until it grows large enough to cause direct invasion or compression of adjacent organs. We report a case of a 77-year-old male presented with dyspnea of exertion and was found to have a large mediastinal mass which was eventually diagnosed as primary mediastinal well-differentiated liposarcoma. The limited respiratory function at the initial presentation prompted phrenic nerve preserving incomplete resection rather than radical removal of the adjacent mediastinal structures. After surgical removal, the recurrence for well-differentiated mediastinal liposarcomas in the mediastinum is unknown; therefore, close follow-up is crucial.

## Background

Liposarcoma is the most commonly diagnosed soft tissue sarcoma in adults but occurs very rarely in the mediastinum [1]. The tumors are often asymptomatic until large which causes symptoms from direct invasion or compression of the heart, great vessels or lungs [2]. Gross examination cannot distinguish between lipoma and liposarcoma. In the majority of cases, computed tomography (CT) and/or magnetic resonance imaging (MRI) are sufficient to determine the nature of the tumor [3-6]. The behavior of any liposarcoma is dependent on its histological subtype. The treatment principles are the same as for other soft tissue sarcomas including surgical removal with adjuvant radiation and chemotherapy [7-9]. We herein describe a case of liposarcoma of mediastinum in a 77-year-old man.

## Case presentation

**Figure 1:**
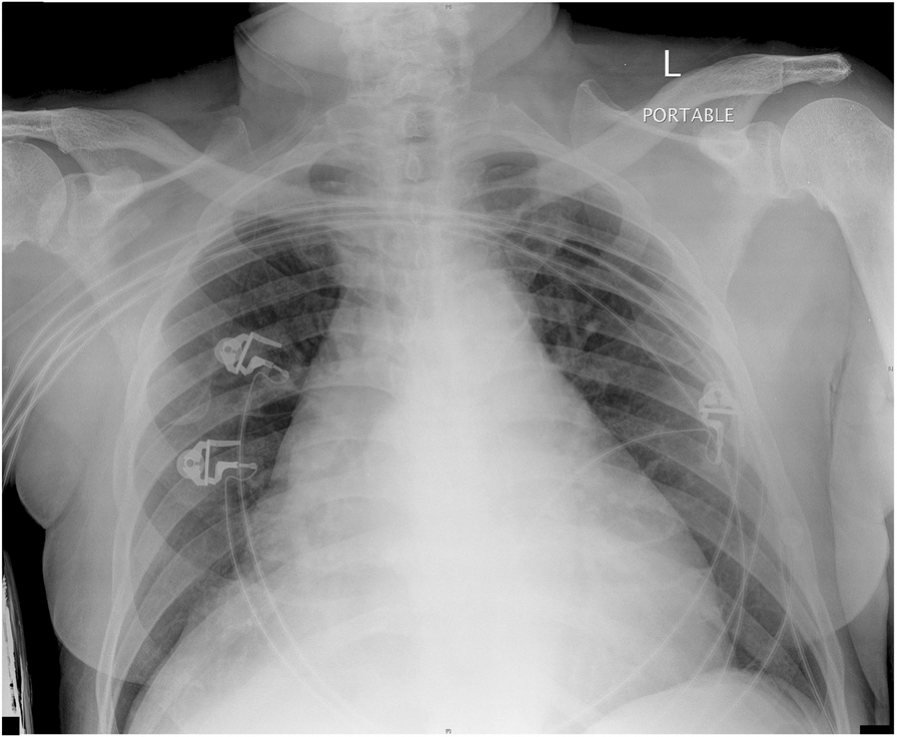
**Preoperative antero-posterior chest radiograph.** Antero-posterior chest radiograph shows widened mediastinum and parenchymal haziness at the bases of both lungs.

**Figure 2:**
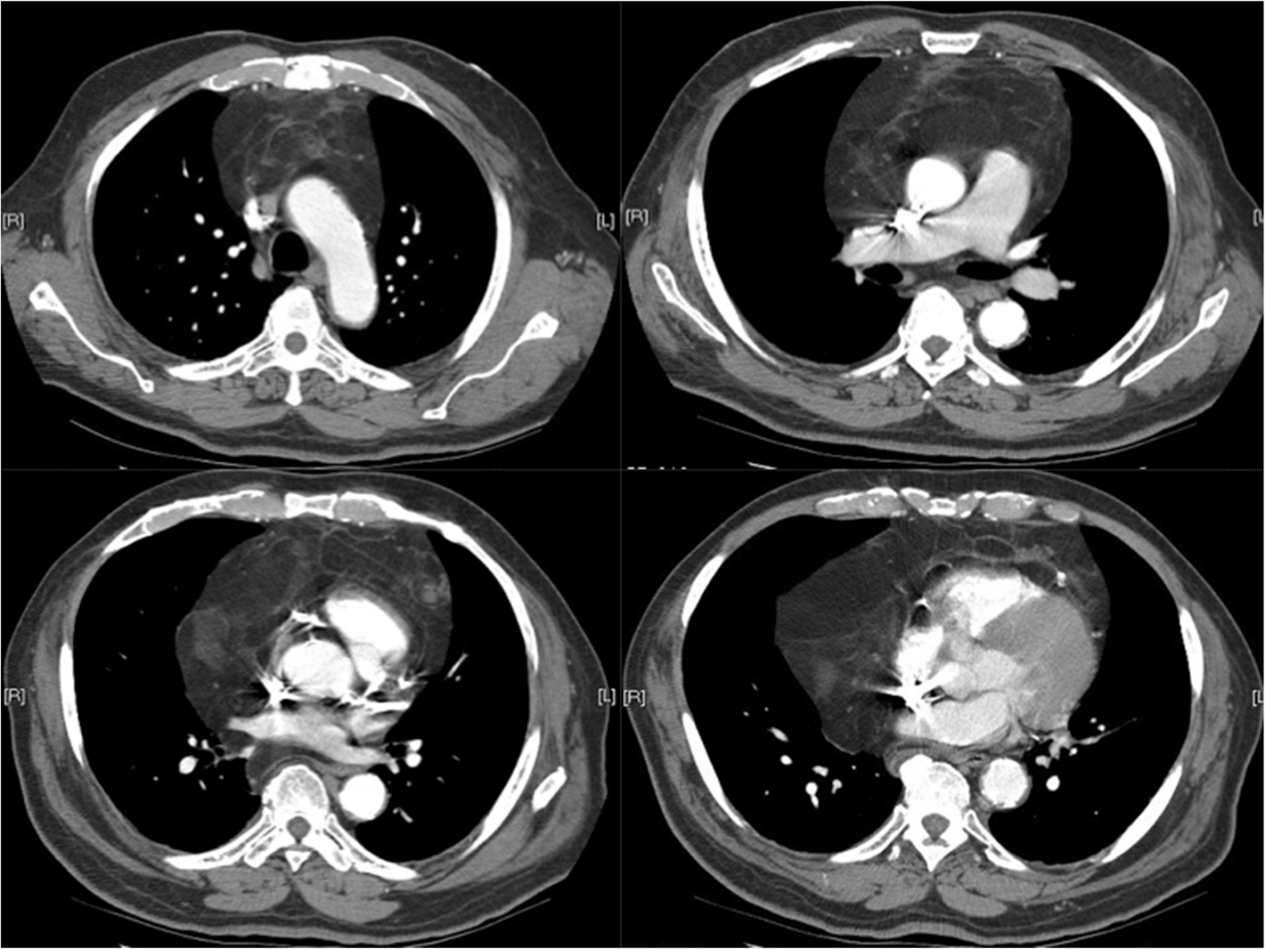
**Preoperative computed tomography scan.** Chest computed tomography scan at the level of pulmonary hilum (upper left image) demonstrates the multi-lobulated mass, well-defined, homogenous, low-attenuation mass in the anterior mediastinum. The other three images show lobulated mass with multiple calcific septations. The fat plane between the mass and the left pulmonary artery is focally obliterated.

**Figure 3:**
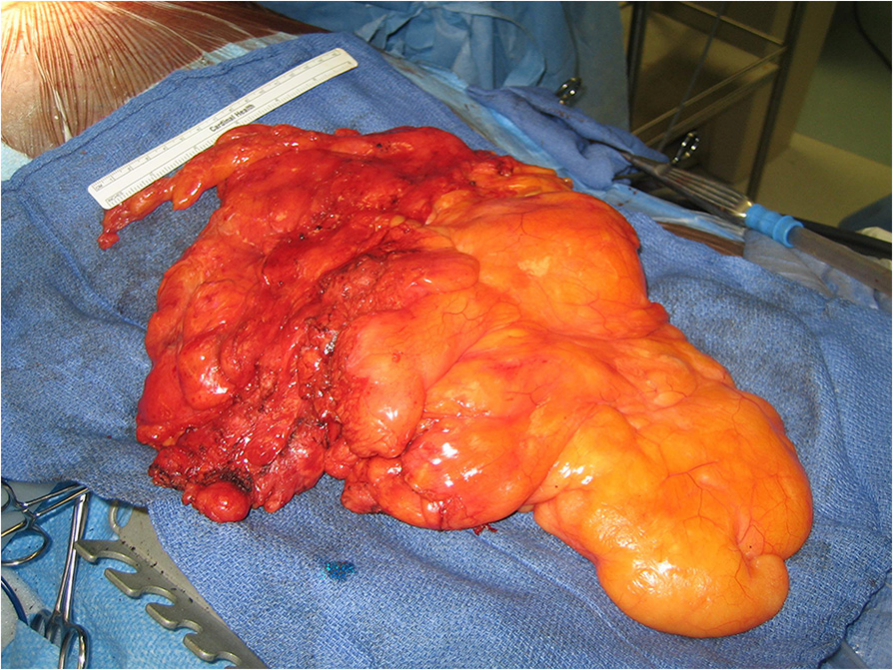
**Intraoperative photograph.** Intraoperative specimen, liposarcoma appearance is similar to that of mature fat.

**Figure 4:**
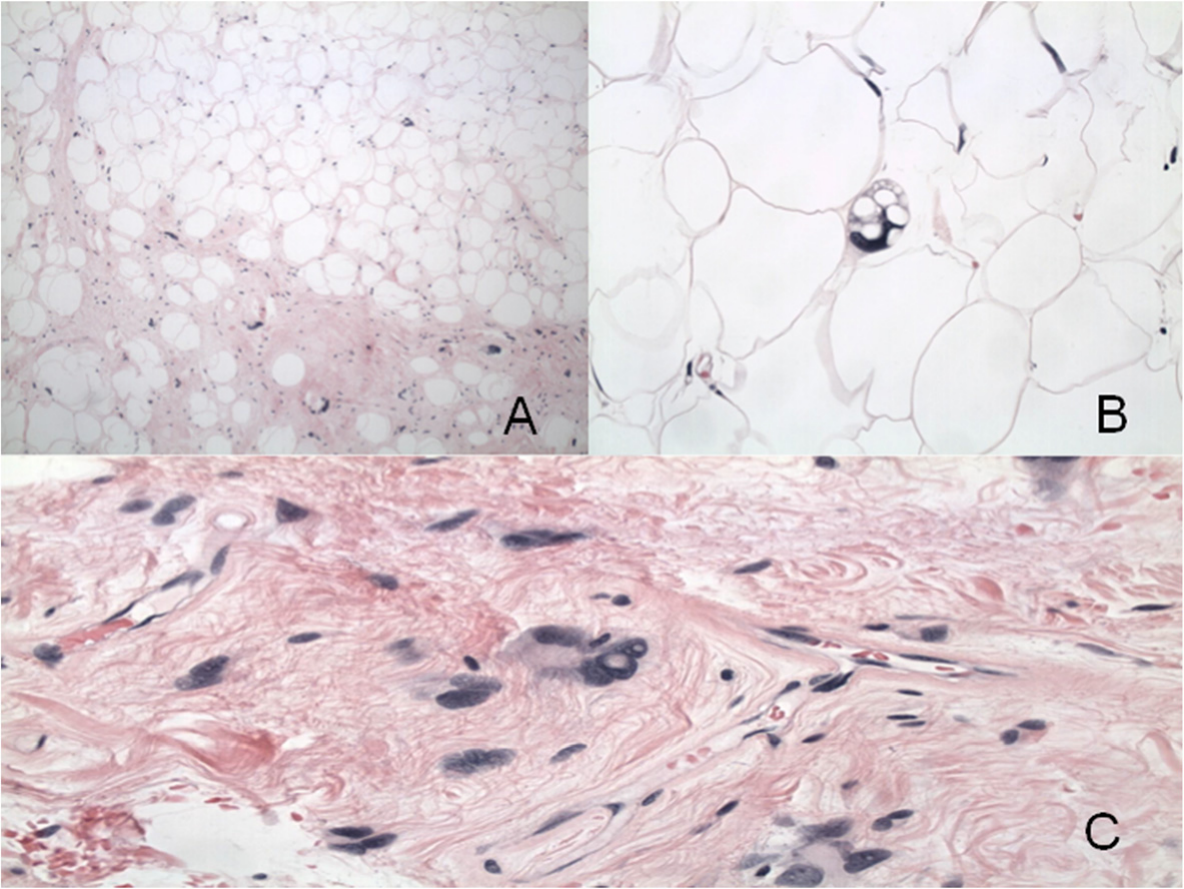
**Histological sections. A**, Adipose cells and fibrous strands with scattered “signet-ring” type cells resembling normal adipose tissue but with large, deep-stranding nuclei (hematoxylin-eosin, original magnification × 200). **B**, Lipoblast showing lipid vacuoles and indented nucleus (hematoxylin-eosin, original magnification × 400). **C**, Lochkern vacuole (hematoxylin-eosin, original magnification × 200).

A 77-year-old Hispanic male presented with a six months history of exertional dyspnea and recent onset of chest pain. He denied fevers or weight loss. His vital signs were normal. Physical examination demonstrated dullness on percussion and decreased breath sounds at the bases bilaterally. Forced expiratory volume in 1 second (FEV1) was 1.78 L and forced vital capacity (FVC) was 2.29 L. Laboratory data, electrocardiogram and arterial blood gas analyses were normal. Chest radiograph demonstrated a widened mediastinum and bilateral lower lobe haziness (Figure 1). A chest CT showed a well-defined, lobulated, heterogenous mass of low attenuation in the anterior mediastinum. The mass abutted the ascending aorta, pulomonary artery, right hemidiaphragm, and the pericardium with focal obliteration of fat planes (Figure 2). Patient refused MRI, but the bone scan, head and abdominal CT scans showed no other lesions. The patient underwent median sternotomy and en block resection of the tumor. An extremely large, well-demarcated and multi-lobulated mass was resected superiorly along the trachea, laterally to both phrenic nerves and inferiorly to both hemi-diaphragms. Both phrenic nerves were preserved with minimal surrounding tissue. The pericardium was intact with no invasion of the heart or great vessels. The tumor measured 36 × 20 × 6.5 cm and weighed 2930 g (Figure 3). Macroscopically, the mass was soft and pale to dark yellow in color, well-circumscribed with rubbery nodules within the specimen. Histologically, the tissue was composed of variable sized mature lipocytes (Figure 4A) with large sharply outlined vaculoles (Lochkern vacuoles), fibromyxoid stroma containing spindle cells (Figure 4C) and lipoblasts with mitotic rate of 1 to 3 per 10 high-power fields (Figure 4B). Final pathological diagnosis was primary mediastinal well-differentiated liposarcoma without extra-mediastinal foci of tumor. Patient was discharged home 8 days after the surgery. Radiotherapy was recommended; however, our patient declined. He was asymptomatic two years after discharge and undergoes surveillance CT of the chest yearly.

### Discussion

Liposarcoma most commonly occurs in the lower extremities and retroperitoneum and rarely in the subcutaneous tissue, paratesticular fat tissue and the mediastinum [1]. Most patients with liposarcomas present complaining of a large mass, often painless unless some sort of trauma has occurred or a vital organ is compressed [2]. Common presenting symptoms of mediastinal liposarcomas are chest pain, dyspnea, wheezing, cough and weight loss [10]. Pericardial and pleural effusions are common, although localized lung collapse is characteristic clinical sign when it reaches larger size [10]. Factors that suggest malignancy are masses greater than 5 cm and lesions that are deep-seated, firm and fixed to underlying structures [1]. Mediastinal liposarcomas are more often found in the anterior mediastinum, adherent to the pericardium and tend to involve the diaphragm and phrenic nerves [11,12]. Esophagoscopy and bronchoscopy may reveal an extrinsic compression effect of the tumor, but no evidence of an intraluminal lesion. In cases where metastatic disease is suspected a transthoracic CT-guided core-needle biopsy or mediastinoscopy is indicated to obtain tissue specimen to establish the diagnosis [12].

Findings in plain chest radiography are nonspecific, and offer little except confirming the presence of a soft-tissue mass. CT provides valuable morphologic information and helps in the differentiation of various types of lipomatous tumors [1-6,10-12]. The typical liposarcoma appearance on CT and MRI is a heterogenic mass composed of adipose tissue mixed with non-adipose components. Radiographic features suggesting higher grade malignancies are large lesion size, presence of thick septations and nodular and/or globular non-adipose mass like areas, and decrease percentage of fat [6]. Well-differentiated liposarcomas are typically composed of more than 75% fat, while the other histologic types usually have less than 25% [6]. Liposarcomas are radiosensitive soft-tissue tumors and the more aggressive tumors demonstrate more radiopacity [5]. Poorly differentiated liposarcomas, particularly the pleomorphic type liposarcoma, may be indistinguishable from other mediastinal malignancies such as malignant fibrous histiocytoma, leiomyosarcoma, desmoids, mesothelial tumors, lymphomas, metastases, and inflammatory masses.

The pathological evaluation is the most important step to differentiate between mediastinal liposarcoma and all other mediastinal fat-containing tumors: lipoma, thymolipoma, teratoma, lymphoma, germ cell tumor, or even herniated peritoneal fat. The World Health Organization currently recognized four subtypes of liposarcoma: well-differentiated, myxoid, pleomorphic and dedifferentiated (Table 1) [13]. Well-differentiated liposarcomas have histologic features resembling mature adipose tissue, as the presence of fibrous tissue septa and spindle cells is the main distinctive pathologic characteristic between them [14]. Liposarcomas are locally aggressive tumors composed of enlarged adipocytes (“signet-ring” type cells) with atypical hyperchromatic cells with angular nuclei and lipoblasts [15]. Overall the cellularity is low and mitotic figures are uncommon, with rare heterologous differentiation and none or only a few lipoblasts. Macroscopically well-differentiated liposarcomas also resemble lipomas, but tend to be larger, often traversed by dense bands of collagen, gelatinous areas, and nodularity with greater variation in size and firmness than an ordinary lipoma. Well-differentiated liposarcomas are associated with abnormalities derived from the q13–15 region of chromosome 12 [1]. Perhaps the best characterized genetic association is in myxoid liposarcoma and represents a translocation or sharing of genetic material between chromosomes 12 and 16. The result is a gene called TLS-CHOP which is an oncogene, or gene that when expressed can lead to the formation of cancer. This particular translocation and its products are specific for myxoid liposarcoma and therefore are diagnostic of this tumor [16].

**Table 1:** Liposarcoma subtypes

	
Well-differentiated	Most common subtype (50% of liposarcomas)
	Mature adipocytes with large fatty vacuoles
	None of a few lipoblasts
	Infrequent mitosis
	Variable myxomatous tissue with occasional dense hyaline sclerosis
	Occasional spindle cells
	Low grade with risk of dedifferentiation
Myxoid	Most common type in pediatric age group
	Immature mesenchymal giant cells in prominent mucopolysaccharide rich stroma
	Lipoblasts with mitotic figures
	Prominent vascularization of branching capillaries (chicken wire pattern)
	Includes round-cell variant as its high-grade counterpart
	Intermediate grade with metastatic risk especially in round-cell variant
Pleomorphic	Rarest type (5–10% of liposarcomas)
	Many lipoblasts with high mitotic rate
	Highly anaplastic sarcoma
	High grade with high risk of local recurrence and metastasis
	May mimic carcinoma or melanoma
Dedifferentiated	Most common with retroperitoneal lesions
	High grade with very hight risk of metastasis

While many of the principles governing the evaluation and diagnosis of soft tissue sarcomas certainly apply to liposarcoma, there are features unique for the treatment of anterior mediastinal liposarcoma that deserve special consideration. Particularly important is the tumor proximity to the vital mediastinal organs: heart, aorta, lungs, superior vena cava, phrenic nerves and diaphragm. The current standard of treatment is surgical removal and perioperative adjuvant radiation and chemotherapy [7-9]. Surgical procedures can be intralesional (within the tumor mass, often leaving gross tumor), marginal (through the surrounding fibrous membrane, often leaving microscopic foci of tumor), wide (outside the membrane and compartment, leaving no tumor other than “skip metastases”), and radical (most often involving the entire limb and including the entire compartment in which the tumor was located). In the anterior mediastinum radical and wide resections are frequently unattainable therefore marginal surgery is acceptable for these lesions. The amount of radiation given to a patient may range from 40 to 60 Gy or more, depending on the extent of the surgery, the anatomic site, and likelihood of microscopic or macroscopic retention of diseased tissue [7]. Local control rates of 85–90% have been achieved with combination therapy of surgery and radiation [8]. The role of chemotherapy in the treatment of liposarcoma is controversial. The principal agents are ifosfamide and doxorubicin, both of which are particularly effective for high-grade tumors [9]. When treated with surgery and perioperative radiation therapy, well-differentiated liposarcomas exhibit a < 10% local recurrence rate and a virtually 0% rate of metastasis. In contrast, pleomorphic liposarcomas recur in about 1/3 of cases and spread in about 40%. Five and ten year survival rates for patients with liposarcomas have been reported as 100% and 87% for well-differentiated, 88% and 76% for myxoid variants and 56% and 39% in the pleomorphic subtype [8,15]. In some patients because of extensive disease, poor clinical status, and comorbidities, despite a favorable pathological diagnosis, no complete curative treatment can be offered. In our patient, we chose to perform marginal resection to spare the phrenic nerves in an attempt to preserve patient’s already compromised respiratory function. Leaving behind microscopic tumor tissue increases the chances of recurrence.

## Conclusion

We reported a case with a large mediastinal mass. Our patient had many of the radiological signs for higher grade malignancy: enormous size, multinodularity with thick calcified septation, but his final histocytological diagnosis was well-differentiated liposarcoma, which has a more benign clinical course. After surgical removal, the recurrence for well-differentiated mediastinal liposarcomas in the mediastinum is unknown; therefore, close follow-up is mandatory.

## Consent

Written informed consent was obtained from the patient for publication of this case report. A copy of written consent is available for review by the Editors-in Chief of this journal.

## Competing interests

The authors declare that they have no competing interests.

## Authors’ contributions

SF and KD wrote the manuscript. KD, CG, DH and RT performed surgery. WK and RT supervised manuscript and entire treatment. All authors read and approved the final manuscript.
